# Structural 3D Domain Reconstruction of the RNA Genome from Viruses with Secondary Structure Models

**DOI:** 10.3390/v13081555

**Published:** 2021-08-06

**Authors:** Simón Poblete, Horacio V. Guzman

**Affiliations:** 1 Instituto de Ciencias Físicas y Matemáticas, Universidad Austral de Chile, Valdivia 5091000, Chile; 2 Chile and Computational Biology Lab, Fundación Ciencia & Vida, Santiago 7780272, Chile; 3 Department of Theoretical Physics, Jožef Stefan Institute, SI-1000 Ljubljana, Slovenia

**Keywords:** RNA secondary structure, RNA tertiary structure, RNA genome, STMV

## Abstract

Three-dimensional RNA domain reconstruction is important for the assembly, disassembly and delivery functionalities of a packed proteinaceus capsid. However, to date, the self-association of RNA molecules is still an open problem. Recent chemical probing reports provide, with high reliability, the secondary structure of diverse RNA ensembles, such as those of viral genomes. Here, we present a method for reconstructing the complete 3D structure of RNA genomes, which combines a coarse-grained model with a subdomain composition scheme to obtain the entire genome inside proteinaceus capsids based on secondary structures from experimental techniques. Despite the amount of sampling involved in the folded and also unfolded RNA molecules, advanced microscope techniques can provide points of anchoring, which enhance our model to include interactions between capsid pentamers and RNA subdomains. To test our method, we tackle the satellite tobacco mosaic virus (STMV) genome, which has been widely studied by both experimental and computational communities. We provide not only a methodology to structurally analyze the tertiary conformations of the RNA genome inside capsids, but a flexible platform that allows the easy implementation of features/descriptors coming from both theoretical and experimental approaches.

## 1. Introduction

RNA genome is an essential part of RNA viruses. In particular, non-enveloped ones convey a direct relationship between capsid proteins and RNA molecules, which was the genesis of several research routes on the assembly, disassembly and delivery functionalities of RNA inside proteinaceus capsids [1,2,3,4,5,6,7,8,9,10,11,12]. RNA is a polyelectrolyte with highly versatile conformations; with self-associating base pairs, it creates a variety of three-dimensional circuitous structural motifs. The organization of the RNA genome is important for many aspects of virus assembly, particularly in steps where different regions of the genome, such as stems and loops, are thought to interact/bind with capsid proteins. However, despite the relevance of its structural organization, there is little known about how it is acquired: first, the formation of an unambiguous secondary structure as patterns of base pairs, followed by the more challenging question of how the tertiary structure is formed [13,14,15,16,17,18,19,20]. In recent years, several chemical probing tools [21,22,23,24,25,26] have tackled possible ways to measure/detect RNA secondary structures; nowadays, they offer (with a reasonable probability) very sophisticated schemes able to propose whole genomes packed inside virus capsids [27], cells [23], among other biological systems [20]. Those results provide valuable information of the secondary structures, which shall be used as a starting point for further computational reconstruction of three-dimensional structures [19,28,29,30]. The latter constitutes an even more challenging task by considering the conformational softness of RNA molecules and its direct implications in the structural heterogeneity of RNA interactions. Thus, the interactions and binding between capsid proteins inside its biological environment can be drastically affected. In this context, besides the secondary structure provided by chemical probing methods, collecting further microscopic information from biophysical techniques [20,31] is crucial to interpret and improve 3D RNA computational modeling. Here, we propose a method that combines a coarse-grained model of RNA with a subdomain composition scheme to obtain the entire genome inside and outside proteinaceus capsids based on secondary structures from chemical probing and structural refinement by biophysical methods (X-ray crystallography, cryoelectron microscopy—CryoEM—among others). Our approach allows the rearrangement of large domains (in the order of hundreds of nucleotides) by using Steered Monte Carlo (SMC) simulations, which include a parsimonious supervision scheme, to generate three-dimensional models from the sole knowledge of the sequence and secondary structure. We tested our method with an extensively studied system, namely, the genome of the STMV. The latter was computationally tackled via molecular simulations; hence, specific structural microscopic arrangements can be also tested for the sake of evaluating our method feasibility [32,33,34,35].

The article comprises several sections, starting with a short description of the method, including the steps for the subdomain reconstruction process. Subsequently, we test the method with the input from the STMV secondary structures proposed by Weeks and co-workers [27], namely, in virio and in vitro models. In a further section, we tackle a particular subdomain of the STMV genome to locally disambiguate between the two most likely structures around a five-fold rotational axis, and briefly discuss the quantities to observe in order to distinguish the optimal structural arrangement. This work concludes with a discussion on how to further couple the present method with computational/theoretical and experimental approaches, and how to address possible issues in the reconstruction of large RNA structures.

## 2. Materials and Methods

### 2.1. Coarse-Grained Model

The SPlit and conQueR model (SPQR) is a coarse-grained representation [36] of RNA, originally designed for structure prediction [37]. Under this scheme, each nucleotide is treated as an anisotropic particle and a spherical bead, which stand for the nucleoside and the phosphate group respectively. The interactions among the coarse-grained nucleotides consist of excluded volume, canonical and non-canonical base pairs, stacking and base-phosphate interactions, with a backbone connectivity that specifies the sugar pucker and the glycosidic bond angle conformation, whereby the excluded volume interaction corresponds to an energy term that prevents the overlap between different coarse-grained beads. In the present work, however, we start with a reduced interaction set, which consists solely of the excluded volume and backbone connectivity, in order to reduce the simulation time, due to the system size. This model [37] has its own energy units, which is denoted by $\epsilon_{s}$ in the following.

In order to enforce a particular geometry on a set of nucleotides, we perform SMC simulations, which are constant temperature Monte Carlo (MC) simulations with additional energy restraints. These simulations minimize the ERMSD metric between the simulated RNA fragment and a target structure: a quantity which has been shown to be suitable for comparing nucleic acids structures [17]. The ERMSD between two structures is defined as a function of the set of vectors { $r_{ij}$} and { $r_{ij}^{r}$}, obtained from the simulated and target structures, respectively. Each of these vectors is calculated from the position of the nucleobase *i* with respect to a reference frame located at the origin of nucleobase *j* with its orientation. The rescaled vectors $\tilde{r}=\left(r_{x}/a,r_{y}/b,r_{z}/c\right)$, with a=b=5 Å and c=3 Å, are used to define the vectors $G\left({\tilde{r}}_{ij}\right)=\;\left(\sin\left(\gamma {\tilde{r}}_{x}/\tilde{r}\right),\sin\left(\gamma {\tilde{r}}_{y}/\tilde{r}\right),\sin\left(\gamma {\tilde{r}}_{z}/\tilde{r}\right),1+\cos\left(\gamma \tilde{r}\right)\right)\Theta \left({\tilde{r}}_{c}-\tilde{r}\right)/\gamma$, where $\gamma =\pi /{\tilde{r}}_{c}$, Θ is the Heaviside function and ${\tilde{r}}_{c}$ is a cutoff parameter. Focusing on a particular fragment denoted by *s*, composed of $N_{s}$ nucleotides, the ERMSD can be written as follows:

(1) $$E_{s}=\sqrt{\frac{1}{N_{s}}\sum_{j,k}{\left|G\left({\tilde{r}}_{jk,s}\right)-G\left({\tilde{r}}_{jk,s}^{r}\right),\right|}^{2}}$$

which allows to define the restraining energy as follows:

(2) $$U_{\mathrm{ss}}=\frac{K_{\mathrm{ss}}}{2}\sum_{s}E_{s}^{2}$$

where $K_{ss}$ is the corresponding harmonic spring constant [17]. In this manner, the relative positions and orientations of the nucleobases belonging to each fragment are enforced to have the values of the target template. In particular, such restraints can be used to impose a secondary structure or to enforce a global structural array between an arbitrary set of nucleotides. This procedure was also used for enforcing other motifs and backmapping RNA structures predicted by SPQR into an all-atom representation, by means of steered-molecular dynamics [37,38]. The value of the ERMSD depends on the adimensional cutoff chosen. Therefore, each result involving the ERMSD specifies this quantity.

An additional restraint can be exerted on the radius of gyration, which has the following harmonic form:

(3) $$U_{RG}=\frac{1}{2}K_{RG}{\left(R_{G}-R_{G}^{0}\right)}^{2}$$

where $K_{RG}$ is a harmonic spring constant, $R_{G}$ is the radius of gyration and $R_{G}^{0}$ is a target value for this quantity.

Finally, an important feature of the method is the use of soft repulsive or attractive energy terms, which are designed to put away colliding coarse-grained nucleotides or to reestablish broken bonds, which allow to easily relax assembled fragments into a more realistic conformation.

### 2.2. Fragment Assembler

Within our method, the secondary structure is given as an input, where we decompose the structure into two type of subdomains: junctions and stem loops. The latter can be composed of one or more sequential complementary double helices, which can be connected by internal loops or be closed by a hairpin.

**Figure 1:**
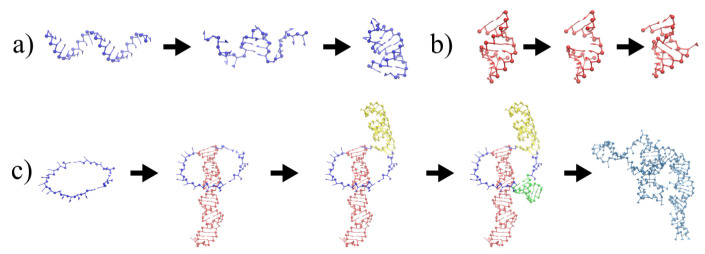
Fragment assembly of STMV genome. (**a**) Reconstruction of a hairpin loop, (**b**) reconstruction of a stem loop. At the end, a short relaxation simulation ensures that the topology is realistic and that no clashes are present. (**c**) Assembly on template junction.

We proceed to generate models for each stem loop as shown in Figure 1a. The hairpins, or whole stem loops, if they are short enough, are designed by applying secondary structure restraints on the closing pairs, starting from a fully extended conformation. This enforcement has to be performed in short steps made out of 3 pairs at a time to avoid the entanglement under the helix formation in the molecule.

Similarly, the size of the stem is increased by exerting the secondary structure restraints on additional unpaired nucleotides, which belong to the hairpin loop. Here, we have empirically determined that the size of such a hairpin loop has to be at most 4 nucleotides. This procedure is necessary in order to prevent the entanglement of the hairpin with itself and with the stem. At the end, the artificially introduced additional pairs are released from their restraint, and the structure is subsequently relaxed with the original secondary structure.

Each of these simulations is performed with temperature *T* such that $k_{B}T=3\epsilon_{s}$ for 100,000 MC sweeps. Each MC sweep consists of a MC trial move on each of the nucleotides.

For larger stem loops, the procedure is illustrated in Figure 1b. A complementary double helix template is created from the first strand of the stem loop. Additional nucleotides are introduced in order to match the number of nucleotides on both strands in the internal loops, if needed. Then, the sequences are replaced by the original one and additional nucleotides are removed. The whole structure is finally relaxed to ensure the connectivity and to enable additional flexibility in the internal loops.

The junctions are generated as ring-like structures, which are composed of strands connected by a closing base pair, where a stem loop is anchored (see Figure 1c). A set of 10 decoys for each junction is generated, simulating by 400,000 MC sweeps at temperature $9\epsilon_{s}/k_{B}T$, under the imposition of a relatively large radius of gyration (between 40 and 90 Å, depending on the system size) and with $K_{RG}=20\epsilon_{s}/$ Å${}^{2}$. Later on, the stems loops are placed on top of their respective closing base-pairs, checking for the possible formation of clashes and knots. Usually, more than one decoy satisfies these conditions, given the extended conformation of the junction ring. This procedure is extremely fast and efficient, given the short size of the junction loops, and it is useful for generating independent geometrical conformations, which are not easy to explore by MC simulations, given the size of the stem loops attached to them in the examined cases. Moreover, given that the in virio structure is likely to be suboptimal in terms of free energy, and that the structural restraints is likely to be applied on the stem loops, it is reasonable to relax and optimize the structure of the junctions in the subsequent step.

Finally, ERMSD restraints can enforce the geometrical arrangement of the multiple stem loops predicted by the secondary structure model. For this, we make use of the X-ray crystal structure of STMV (PDB 4OQ9), where 30 stem loops are reported, comprising approximately 59% of the genome, but without specification of their sequence. Several efforts have been made in this direction, although they did employ different methodologies and different assumptions for secondary structure models [32,33,35].

Note also that our fragment assembly procedure could, in principle, be applied to other RNA coarse-grained models [39,40,41,42,43]; however, the exact details on resolution, energy terms and/or other model parameters have to be individually evaluated and are beyond the scope of this article.

## 3. Results

**Figure 2:**
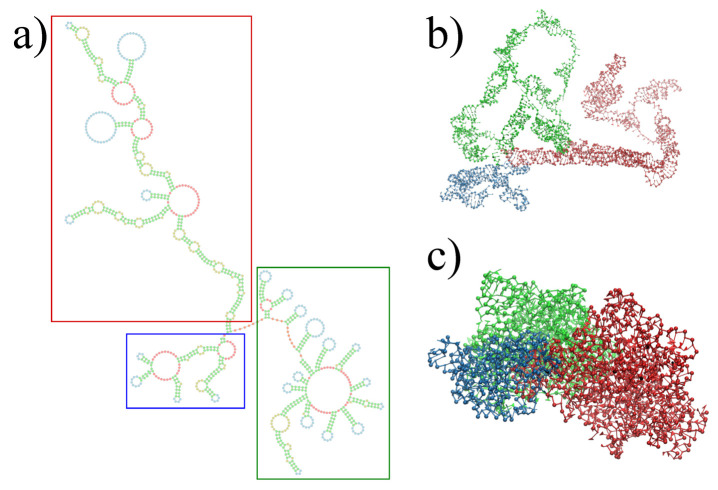
Full reconstruction of the STMV in virio genome. (**a**) Secondary structure proposed by Weeks and co-workers [27], with colored rectangles distinguishing each domain: blue for 5${}^{\prime }$ domain, red for central T domain and green for 3′ domain. (**b**) Reconstruction after assembly and energy minimization and (**c**) after SMC simulation with a minimum radius of gyration.

**Figure 3:**
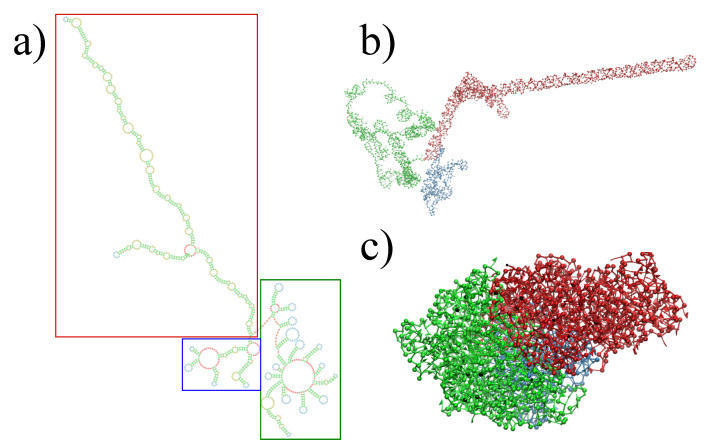
Full reconstruction of the STMV in vitro genome. (**a**) Secondary structure proposed by Weeks and co-workers [27], with colored rectangles distinguishing each domain: blue for 5′ domain, red for central T domain and green for 3′ domain. (**b**) Reconstruction after assembly and energy minimization and (**c**) after SMC simulation with a minimum radius of gyration.

**Figure 4:**
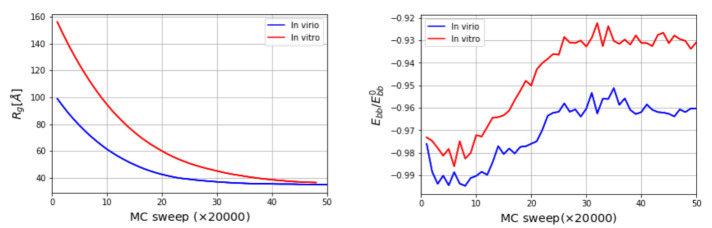
Radius of gyration $R_{g}$ as a function of MC sweep (**left**) and normalized backbone energy (**right**). $E_{bb}^{0}$ corresponds to the backbone energy of a single strand of the same size with a conformation taken from an A-form double helix.

The three-dimensional in virio and in vitro models of the STMV genome are shown in Figure 2 and Figure 3. Each decoy was minimally supervised, obtaining a refined structure with no broken bonds, nor clashes, nor knots between their components. Nevertheless, their extension is far from the dimensions inside a virus, reaching a radius of gyration of 99 Å for the in virio model, which yields a maximum distance of 182 Å between a phosphate group and the center of mass. Clearly, these values are well beyond the radius of the STMV, including its capsid, which is approximately 85 Å. A SMC simulation was performed for minimizing the radius of gyration for 1,000,000 MC sweeps ($R_{G}^{0}=10$ Å, $K_{RG}=200\epsilon_{s}/$Å${}^{2}$), which maintains the secondary structure restraints. We observed that both structures can reach a compact state in a similar amount of time, under the effect of a strong bias on the radius of gyration, as seen in Figure 4. Nevertheless, the backbone energy reaches a higher value for the in vitro structure, which suggests that a higher deformation of the molecule has to take place in order to satisfy the spatial restraint. The ERMSD (with a cutoff of 100), which enforces the secondary structure, increases by around 5% in both cases, which expresses that the structure of the restrained stems is minimally affected.

**Figure 5:**
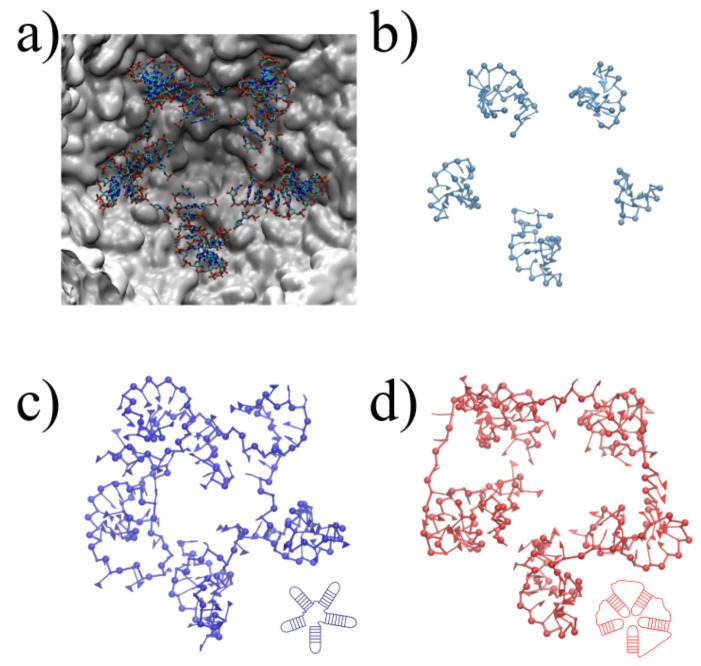
Steered Monte Carlo simulations on five sequential stem loops. (**a**) X-ray structure (PDB:4OQ9) on five-fold symmetry axis, containing 5 stem loops of undefined sequence. (**a**) Stem loop templates for SMC simulation extracted from the PDB and brought to coarse-grained resolution. (**c**,**d**) Final coarse-grained conformations after steering, employing different target orientations consistent with experimental restraints illustrated at the bottom right of each configuration.

**Figure 6:**
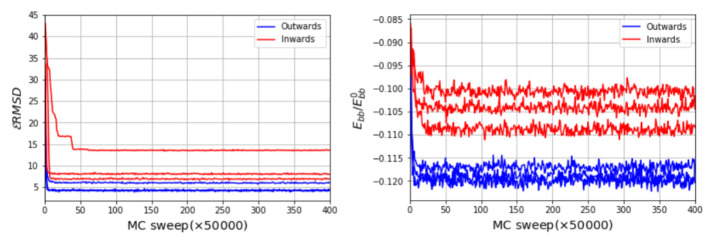
ERMSD as a function of MC sweep for inward and outward scenarios (**left**) and normalized backbone energy (**right**) normalized by a reference single strand in A-form structure. The used ERMSD adimensional cutoff is 20.

An optimal determination of the 3D encapsidated structure of the RNA genome goes beyond the capabilities of the current RNA–protein interaction modeling, which was discussed and conceptualized under different hypotheses, specifically for virus assemblies [44,45,46,47]. Nevertheless, we aim to present a systematic and reasonable model, which brings a new methodology, to structurally interpret, explain and evaluate the feasibility of the chemically probed secondary structures and the further microscopic characteristics of confined RNA inside virus capsids. We illustrate the application of SMC simulations on a fragment of the 3′ domain, which comprises nucleotides 772 to 897. This fragment is composed of five sequential hairpins, which shows resemblance to the secondary structure models proposed earlier [35]. It also serves as an initial benchmark to test the steering around a five-fold symmetry axis, as illustrated in Figure 5a, using the stems determined experimentally as templates for the SMC simulation. Despite the simplicity of this benchmark, note that the arrangement of the structure is not uniquely defined. Such heterogeneity relies mainly on two different scenarios, with the hairpins inward and outward to the pentameric center, as illustrated in Figure 5c,d. Starting from three different initial conformations relaxed after 50,000,000 MC sweeps, we impose the ERMSD restraints to observe a final structure that resembles the template, depicted in Figure 5c. We monitor the backbone energy per nucleotide (Figure 6), in reduced units, where we find that, in fact, the additional stretching present in scenario two (inwards conformation) is well reflected in the backbone energy, regardless of the initial configuration. Moreover, the ERMSD reaches considerably lower values, independently of the initial condition, which states that the inward scenario can become entangled more easily during the simulation. Once the energetically correct scenario under a efficient coarse-grained representation is determined, our method also offers a direct way of backmapping, should the microscopic application demand so.

## 4. Discussion

Our results show a structural 3D domain reconstruction of the RNA genome from viral secondary structures, and propose a way to control the quality of the model based on further microscopic information and/or modeling hypotheses. There are several routes and assumptions involved in the quality control of those 3D reconstructions. Here, we classify them in two main routes, namely, high-resolution microscopic information and novel computational/theoretical models. High-resolution microscopic information, such as cryoelectron microscopy [48,49,50], probe RNA structures inside viruses. On one hand, those advanced microscopy techniques coincide with the ability of encapsidated RNA to form globally more than one structure inside the virus. On the other hand, localized subdomains can form partially ordered structures (with well-defined minimum energy structures), as is the case of the five-fold symmetry example. Nonetheless, many subdomains of such encapsidated RNA lie within the ’dynamic structures’, which are commonly difficult to efficiently sample by pure experiments. Hence, the field appeals to computational models, which have been used, for example, to refine 3D RNA reconstructions with additional restraints on tertiary contacts given by SAXS experiments [51], or the reconstruction of the frameshift stimulating element from SARS-CoV-2 all the way down to atomistic resolution. Such studies were based on chemical probing and CryoEM [52]. Revisiting microscopic information with higher resolution structural information has resolved the formerly intractable structure Tetrahymena ribozyme [53]. The latter successful studies, among others currently in progress, brings us to a particular question: what if 3D domain reconstruction methods could also improve the current shortage of theoretical conceptualization and prediction for particular physical models? For example, in the case of protein shells encapsulating RNA, the precise origin of interactions between RNA and capsid proteins is still unsolved, although several theories have been brought to discussion [47]. To this end, we also prepared our method to be rapidly extended in this direction, especially for coupling diverse types of molecular interactions, such as electrostatic and van der Waals, among others, to candidate 3D reconstructed domains (more details in the following repository https://doi.org/10.5281/zenodo.5035769). Such an approach is complex by itself; however, the offered ability of our method to discern between molecular interactions can be used to test physical assumptions, and guide the interpretation of experiments and possibly the direction of new experiments. On top of this general method improvements, a robust parametrization of the underlying RNA-protein molecular interactions would consequently permit the modification and/or control of the capsid status from assembled to disassembled or vice versa, which are awaited features for further applications in nanomedicine.

## 5. Conclusions

We have presented a new computational method for the 3D reconstruction of the complete RNA genome of virus particles. The RNA genome of the STMV was tested systematically with our algorithm, where we demonstrated the possibility of generating reasonable conformations of the three-dimensional structure of the RNA genome with coarse-grained efficient models (1 CG bead ≈ 30 atoms per nucleotide). This method includes several features, such as the radius of gyration topological constraints, or diverse restraints applied to RNA stems. In addition, our method can be easily extended and combined with further experimental information and/or computational/theoretical models. We have further shown an example of additional microscopic analysis with the five-fold hairpins distribution disambiguation for the STMV, based on the additional X-ray structural information. Finally, we have also discussed examples on how to not only further test the novel microscopic information, but to combine it with theoretical and semi-empirical models to elucidate further mechanisms of virus capsid assembly, disassembly and RNA delivery.

## Data Availability

The data are available at the following Zenodo repository: https://doi.org/10.5281/zenodo.5035769.
